# Multi-residue determination of anticoagulant rodenticides in vertebrate wildlife and domestic animals using Ultra (High) Performance Liquid Chromatography Tandem Mass Spectrometry

**DOI:** 10.1016/j.mex.2018.02.001

**Published:** 2018-02-22

**Authors:** Michael J. Taylor, Anna Giela, Claire Senior, Elizabeth A. Sharp, Christopher M. Titman, Octavio P. Luzardo, Norberto Ruiz Suárez

**Affiliations:** a Chemistry Branch, Science and Advice for Scottish Agriculture (SASA), Roddinglaw Road, Edinburgh, EH12 9FJ, UK; b Shimadzu UK Ltd., Milton Keynes, MK12 5RD, UK; c Toxicology Unit, Research Institute of Biomedical and Health Sciences (IUIBS), Universidad de Las Palmas de Gran Canaria, Paseo Blas Cabrera Felipe s/n, 35016, Las Palmas, Spain; d Spanish Biomedical Research Centre in Physiopathology of Obesity and Nutrition (CIBERObn), Paseo Blas Cabrera Felipe s/n, 35016, Las Palmas, Spain; e Ghent University, Sint-Pietersnieuwstraat 25, B-9000, Ghent, Belgium

**Keywords:** An improved multi-residue method for the determination of 9 anticoagulant rodenticides in liver tissue from non-target vertebrate wildlife and domestic animals using Ultra (High) Performance Liquid Chromatography Tandem Mass Spectrometry (UHPLC–MSMS), Anticoagulant rodenticides, UHPLC–MSMS, multi-residues, GPC clean-up, liver

## Abstract

Anticoagulant rodenticide (AR) products are used globally to control rodent pests in domestic, urban, agricultural and industrial environments. However, there is a substantial volume of evidence that non-target vertebrate wildlife i.e. predators and scavengers in particular and other animals, are vulnerable to contamination via direct or indirect routes of exposure. The determination of multiple AR residues in liver tissue samples that can range from remnants of a small bird of prey liver to an intact liver from a large mammal is complicated as residue levels encountered can vary considerably too. So, the utilisation of ultra-sensitive systems has to be carefully considered in order to allow routine application of the method to all sample compositions presented for analysis. The UHPLC–MSMS method described now:

• permits quantitative analysis of ultra-low levels of multiple-residues (0.0025–1 mg kg^−1^) in a single experiment.
• uses the same U(H)PLC column for the determination of AR and multiple-pesticide residue in similar specimens.
• allows higher sample throughput due to shaking rather than tumbling of samples during the extraction procedure.

## Graphical abstract

**Figure:**
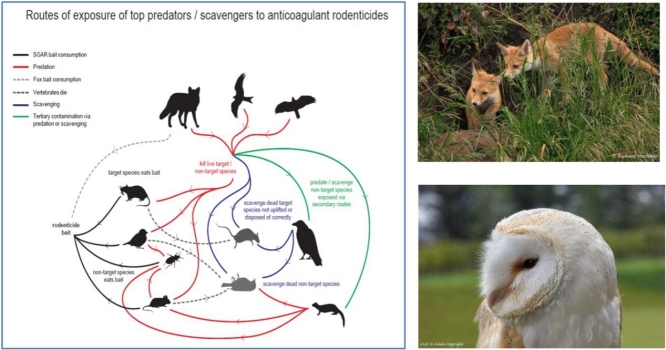

## Method details

### Reagents and preparation of solvent standards

All AR standards were certified reference materials (purity ranging from 98% to 99.5%) and purchased from Dr. Ehrenstorfer (Augsburg, Germany). Solvents used throughout were HPLC-grade unless specified otherwise and supplied by Rathburn Chemicals Ltd. (Walkerburn, Scotland UK). Stock solutions (400 μg ml^−1^) of 8 individual pesticides were prepared using HPLC-grade methanol. Aliquots were taken to compose a standards mixture (5 μg ml^−1^) of brodifacoum, bromadiolone, chlorophacinone, coumatetralyl, difenacoum, diphacinone, flocoumafen and warfarin (standard A). From this and a 10 μg ml^−1^ standard of difethialone (pre-purchased in methanol – standard B), an intermediate solution at 0.4 μg ml^−1^ was prepared by combining 1.6 ml of standard A and 0.8 ml of standard B to a final volume of 20 ml with methanol (solvent standard 8). This intermediate solution was then used to prepare a series of solvent standards (1–7) as detailed below in Table 1.

**Table 1:** Preparation of ‘pure’ solvent standards.

Standard	Volume of standard taken	Rodenticide Conc. (μg ml^−1^)	Final Volume
Solvent std 8 in methanol	1.6 ml Rod Mix (5 μg ml^−1^) and 0.8 ml difethialone (10 μg ml^−1^)	0.4	20 ml

Solvent std 7^a^	5.0 ml solvent std 8	0.2	10 ml
Solvent std 6^a^	2.5 ml solvent std 8	0.1	10 ml
Solvent std 5^a^	2.0 ml solvent std 8	0.04	20 ml
Solvent std 4^a^	5.0 ml solvent std 5	0.02	10 ml
Solvent std 3^a^	1.0 ml solvent std 5	0.004	10 ml
Solvent std 2^a^	0.5 ml solvent std 5	0.002	10 ml
Solvent std 1^a^	0.5 ml solvent std 4	0.001	10 ml

a All made up to volume using 5 mM methanolic ammonium acetate solution.

### Preparation of chicken liver (blank) matrix

50 g portions of ‘chopped’ chicken liver (intended for human consumption and purchased from local retail outlets) were weighed separately into 4 × 1 l beakers. 0.5 g (± 0.01 g) of solid ascorbic acid was added to each beaker and the contents were mixed thoroughly using a glass rod. 500 g of anhydrous sodium sulphate was then added to each beaker and mixed thoroughly in order to absorb moisture.

The contents were allowed to dry for 30 min and mixed again until a friable mixture was obtained. The contents of each beaker was divided equally into 250 ml bottles and 100 ml (± 10 ml) of extraction solvent (1:1 v/v chloroform:acetone plus 0.075% ascorbic acid) was added. The bottles were securely capped and placed on a shaker for at least an hour at 135 strokes per minute. The filtrate and washings from each extraction bottle were collected into 250 ml round-bottomed flasks after being passed through a qualitative filter paper (18.5 cm).

The contents of each 250 ml round bottomed flask were combined and evaporated to dryness using a rotary evaporator with heated bath (IKA, Oxon UK). The bath temperature should not exceed 40 °C. Approximately 20 ml cyclohexane:ethylacetate (1:1 v/v) was used to re-dissolve the residual material with the aid of ultrasonication and transferred to a volumetric flask (100 ml). The final extract was made up to volume with cyclohexane:ethylacetate (1:1 v/v) to give a final matrix concentration of 2 g ml^−1^. The extract is now ready for clean-up using Gel Permeation Chromatography (GPC).

### GPC clean-up

Twenty x 4 ml portions of crude chicken liver extract were filtered into GPC vials which were then sealed and placed into the GPC sample rack. The crude extracts (∼2 ml) were applied to the GPC column and automated GPC clean-up was performed using a Gilson GX-271 Liquid Handler system (Gilson U.K., Luton, UK) and LC Tech column-082 500 × 40 mm, 25 mm, bed length 320 mm, 50 g: (ARC Sciences, Alton UK) in ethylacetate/cyclohexane and the method (Table 2) yielded an elution profile shown in Table 3 below. The solvent mixture employed was cyclohexane:ethylacetate (1:1 v/v)

**Table 2:** GPC clean-up method.

Refill Speed	125 ms
Compressibility	46 M bar ^−1^
Head size	10
**Calibration Mode**	
Flow rate	5.0 + 0.1 ml min^−1^
Injection volume	3000 μl
Number of fractions	35
Collection time	1 min
**Sample Mode**	
Flow rate	5.0 + 0.1 ml min^−1^
Injection volume	3000 μl
Number of fractions	1
Collection time	21 min

**Table 3:** GPC Elution Profile.

AR	Waste (min)	Collect (min)
Brodifacoum	18	13
Bromadiolone	15	10
Chlorophacinone	18	15
Coumatetralyl	18	13
Difenacoum	17	16
Difethialone	19	14
Diphacinone	17	16
Flocoumafen	13	17
Warfarin	16	14

The cleaned-up extracts were combined and evaporated just to dryness by rotary evaporation (bath temperature should not exceed 40 °C). The residue was redissolved with the aid of ultrasonication in 5 mM methanolic ammonium acetate solution (5 ml) and quantitatively transferred to a volumetric flask (20 ml). This gave a final matrix concentration of 4 g ml^−1^.

A separate experiment was and is conducted to check the validity of the ‘rodenticide-free’ chicken liver before it was/is used to prepare matrix-matched standards and spikes.

### Preparation of matrix-matched standards

Matrix-matched standards were prepared as follows: Each solvent standard (1–8: Table 1) was diluted 2-fold into 5 ml volumetric flask that contained 0.25 ml of chicken liver matrix solution. They were made up to volume using 5 mM methanolic ammonium acetate solution in order to produce the following range of matrix-matched standards: 0.0005 μg ml^−1^, 0.001 μg ml^−1^, 0.002 μg ml^−1^, 0.01 μg ml^−1^, 0.02 μg ml^−1^, 0.05 μg ml^−1^, 0.1 μg ml^−1^ and 0.2 μg ml^−1^ (final matrix concentration ≡0.2 g ml^−1)^). Both, the matrix-matched and the solvent standards were prepared every 7 days to ensure the correct quantification of samples.

### Sample preparation and clean-up

Liver tissue was finely chopped and a portion (≤ 4 g) was weighed into a beaker (100 ml) then 40 ± 1 mg of solid ascorbic acid was added and mixed thoroughly using a glass rod. Sufficient amount of anhydrous sodium sulphate was added to absorb moisture. The mixture was left to dry for 20–30 min until friable then transferred into an extraction bottle (250 ml) and 100 ± 10 ml of extraction solvent was added. The bottle was securely capped and placed on a shaker for at least an hour at 135 strokes per minute. The crude extract was filtered off through a qualitative filter paper (18.5 cm) with washings into a round bottom flask (150 ml) and evaporated just to dryness by rotary evaporation (bath temperature not exceeding 40 °C). The dry residue was re-dissolved in approximately 2 ml of cyclohexane/ethyl acetate (1:1 v/v) and the resulting extract was transferred quantitatively to a volumetric flask (4 ml) and made up to volume with the same solvent mixture.

Liver tissue extracts were filtered through glass fibre syringe filters (25 mm, 1.2 μm) and 2 ml applied to the GPC column (approx. 2 g of extract). The first 60 ml of eluate were discarded and the next 100 ml collected. The cleaned-up extract was evaporated just to dryness using a rotary evaporator (bath temperature not exceeding 40 °C) and re-dissolved, with the aid of ultrasonication in 5 mM methanolic ammonium acetate solution (10 ml) for analysis by UHPLC–MSMS. It is not unusual for the available sample weight to be <<<4 g. Therefore it is often necessary to adjust the final volume of 5 mM methanolic ammonium acetate used in order to maintain a matrix concentration of 0.2 g ml^−1^.

### Preparation of fortified liver matrix AQC samples (spikes)

Blank liver tissue samples were fortified, prior to extraction, to generate 6 liver spikes at 0.005 mg kg^−1^, 6 liver spikes at 0.02 mg kg^−1^ and 6 liver spikes at 0.1 mg kg^−1^. Three spike solutions were prepared as follows:

• Spike solution 1: 0.4 μgml^−1^: 1.6 ml of standard A (5 μg ml^−1^) and 0.8 ml of standard B (10 μg ml^−1^) into 20 ml volumetric flask
• Spike solution 2: 0.08 μg ml^−1^: 4 ml of Spike solution 1 into 20 ml volumetric flask
• Spike solution 3: 0.02 μgml^−1^: 1 ml of Spike solution 1 into 20 ml volumetric flask

Then, 1 ml of spike solution 3 0.02 μg ml^−1^ was added to 6 blank liver samples (4 g) to generate spikes at 0.005 mg kg^−1^. 1 ml of spike solution 2 0.08 μg ml^−1^ and 1 ml of spike solution 1 0.4 μg ml^−1^ were used to generate spike samples at 0.02 mg kg^−1^ and 0.1 mg kg^−1^ respectively. All spiked samples were extracted following the ‘Sample preparation and clean-up’ protocol.

### UHPLC–MSMS

UHPLC–MSMS was achieved using a Nexera X2 UHPLC system coupled to a LCMS-8050 triple quadrupole mass spectrometer (Shimadzu Corporation, Japan). The chromatographic separation was performed using a Kinetex C18 50 × 4.6 mm, 2.6 μm analytical column (Phenomenex, Macclesfield, UK) maintained at 40 °C. Mobile phases were (A) water/methanol 95/5 v/v, 5 mM ammonium acetate, and (B) methanol, 5 mM ammonium acetate. The flow was set at 0.4 ml min^−1^ and the volume injected was 3 μl. The total run time was 6 min and the gradient was programmed as follows: 0 min, 10% B; 0.3 min, 40% B; 3.1 min, 98% B; 4.1 min, 98% B; 4.2 to 6.00 min, equilibration time. Retention times of each compound were initially determined in the MRM data acquisition and negative ionisation mode following assignment of the corresponding molecular anion species.

Analyses were performed using electrospray ionisation (ESI) in negative ionisation mode using a Dual Ionisation Source (DUIS). A pause time of 2 ms and dwell time 5–10 ms were used. Argon of 99.9% purity (BOC Manchester, UK) was used as collision gas (270 kPa cell pressure). A combined air and nitrogen generator (Peak Scientific, Renfrew, UK) was used to supply nitrogen as the drying and nebulizing gas, and air as the heating gas, set at universally applied values of 10 l min^−1^, 2 l min^−1^ and 10 l min^−1^, respectively. The interface temperature was 300 °C, the DL (Desolvation Line Assembly) temperature held at 250 °C and heating block temperature was 400 °C. The DUIS interface and corona needle voltages (ESI negative mode) were maintained at −3.00 kV and −3.50 kV, respectively. The UPHLC–MSMS system was controlled and the data acquired and processed using ‘Labsolutions’ software. Data were processed using ‘Labsolutions Insight’ software (Shimadzu Corporation, Japan).

The optimum multiple reaction monitoring (MRM) transitions were determined for each analyte by flow-injection analysis of methanolic solutions of the individual solvent standard directly into the ionisation source. Optimum collision energy values were determined for each analyte/MRM transition. The precursor ion → product ion (MRM) transitions listed in Table 4 were used for construction of the associated calibration curves and subsequent quantitative screening and confirmation of residues in quality control samples and real samples.

**Table 4:** AR structures, formulae, precursor → product ion (MRM) transitions and ionisation parameters (Electrospray: negative ion mode).


AR	M	Ion	MRM *screen*	CE	MRM *confirmation*	CE	RT
Warfarin	308	[M−H]^−^	307.1 > 250.05	23	307.1 > 161.10	20	2.75
Coumatetralyl	292	[M−H]^−^	291.3 > 141.15	27	291.3 > 247.10	23	2.79
Diphacinone	340	[M−H]^−^	339.1 > 167.15	24	339.1 > 116.10	45	3.15
Chlorophacinone	374	[M−H]^−^	373.1 > 201.10	23	373.1 > 145.05	23	3.48
Bromadiolone	526	[M−H]^−^	525.2 > 250.10	37	525.2 > 181.15	36	3.72
Difenacoum	444	[M−H]^−^	443.3 > 293.15	34	443.3 > 135.25	36	3.86
Flocoumafen	542	[M−H]^−^	541.3 > 382.15	26	541.3 > 161.05	35	3.98
Brodifacoum	522	[M−H]^−^	521.2 > 135.10	38	521.2 > 143.10	53	4.10
Difethialone	538	[M−H]^−^	537.2 > 151.10	38	537.2 > 371.00	36	4.13

M = Nominal Mass; CE = Collision Energy (v): RT = Retention Time (mins).

### Validation and analytical quality control

In order to assess the dynamic range of quantitation of each AR in this UHPLC–MSMS system, we investigated (i) the linearity of calibration over the range of residue concentrations, (ii) accuracy and limitations of quantitation using quadratic calibration and (iii) opportunity to eliminate sample dilution and repeat analyses.

**Fig. 1:**
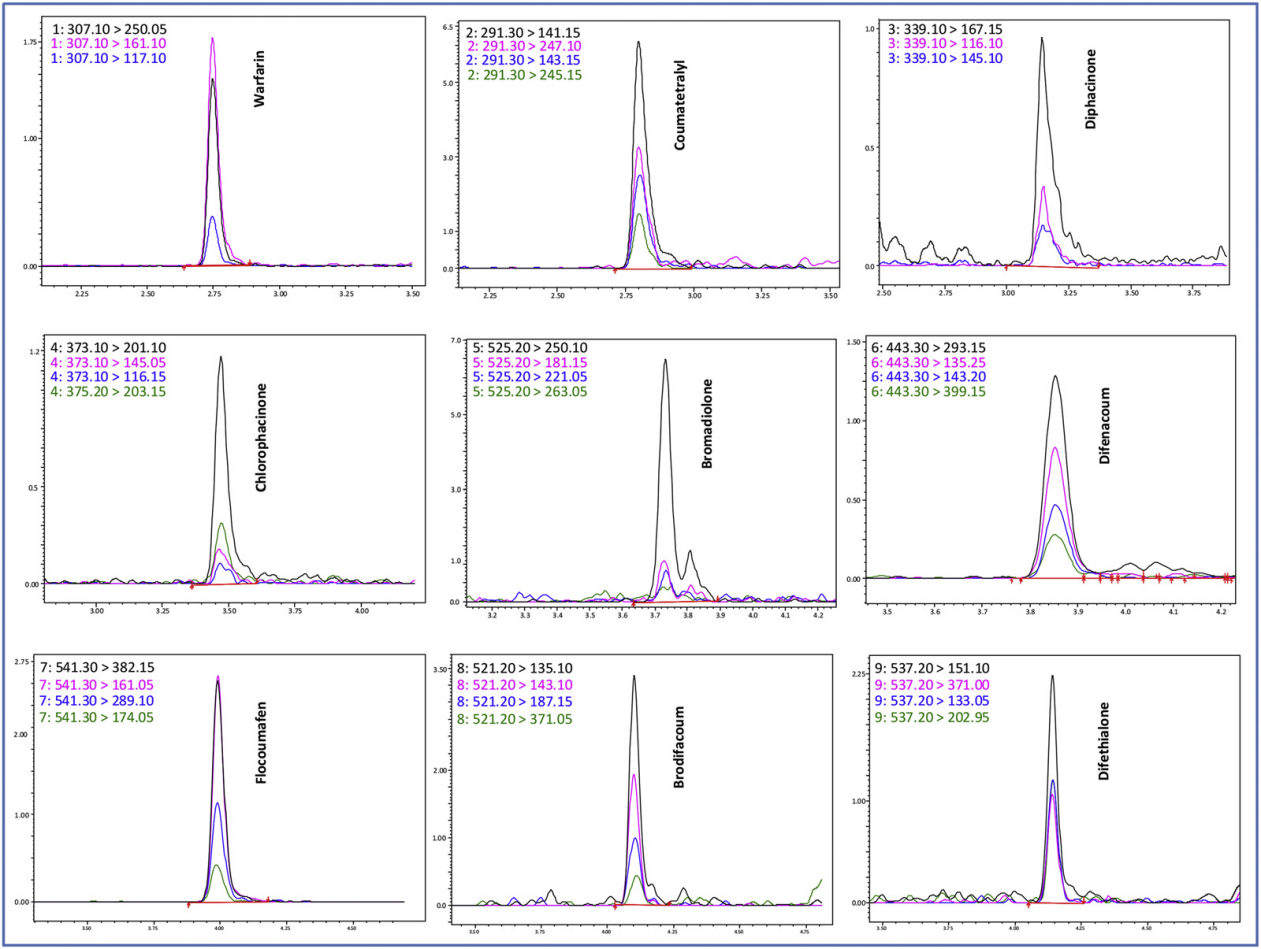
AR/MRM ion chromatograms yielded by 0.001 μgml^−1^ matrix-matched standard. (For interpretation of the references to colour in this figure legend, the reader is referred to the web version of this article.)

This was achieved following the use of multiple AR/MRM transitions i.e. of varying relative intensity, to generate corresponding calibration curves. At least 3 MRMs per AR were identified and processed (Fig. 1). Each AR/MRM combination selected yielded quadratic calibration curves over a residue concentration range covering 3-orders of magnitude (0.0005–0.5 μgml^−1^) and this was generally irrespective of the relative intensity of the selected MRM.

However, quantitation of some AR standards at levels close to the lowest calibration level (LCL) was not reliable using the above range of calibration standards and quadratic calibration. Consequently, it was practical and appropriate to routinely generate restricted ‘low-level’ 5-point linear calibration curves i.e. 0.025–0.1 mg kg^−1^ since this covered the range of AR residues most commonly detected and encountered [[1], [2]]. However, data from the extended (8-point) range of calibration standards was also collected. Calibration was acceptable if the correlation coefficient (R^2^) values ≥0.96 i.e. for either fit [3].

The procedure was subsequently validated following a series of experiments whereby a minimum of 5 replicate spikes at three different levels were analysed to generate mean recovery values and to set a limit of quantitation (LoQ). The LoQ was set when a signal to noise ratio of ≥3:1 (peak to peak) was achieved for the lowest calibration level. The two most intense MRM transitions (screen and confirmation – Table 4) were monitored for each AR. Recovery values were deemed acceptable if they fell within the range 60%–140%, yielded a mean value between 70%–90% and a corresponding co-efficient of variation (CV%) ≤20%. Retention Time tolerance was set at ± 0.1 min and the ion ratio limit was ± 30% difference i.e. in accordance with SANTE AQC and validation guidelines for multiple pesticide residues analysis in food and feed [3]. Table 5 contains validation data obtained for 7 out of 9 ARs as recoveries for chlorophacinone and diphacinone were erratic and the method therefore deemed qualitative for these two ARs. The LoQ was determined and set at 0.003 mg kg^−1^ (≡ 0.0005 μg ml^−1^) for 7/9 ARs. The uncertainty of measurement for the liver tissue validation data i.e. expanded uncertainty = 21% [4]. This was determined from validation experiments conducted on different days and by different analysts. The dataset used to determine and refresh the expanded uncertainty values is augmented by incorporation of AQC data from successive and longer term experimental batches.

**Table 5:** Method Performance Characteristics: Recoveries and CVs in fortified liver tissue.

	Fortification Level mgkg^−1^					
AR	0.1 (n = 6)		0.02 (n = 6)		0.005 (n = 5)	
	Mean	CV%	Mean	CV%	Mean	CV%
Warfarin *screen*	106	6	105	4	100	7
Warfarin *conf*	106	6	105	5	99	6
Coumatetralyl *screen*	105	5	107	4	100	3
Coumatetralyl *conf*	107	5	106	5	104	3
Bromadiolone *screen*	88	3	85	9	76	8
Bromadiolone *conf*	89	5	83	6	77	18
Difenacoum *screen*	91	3	86	7	77	9
Difenacoum *conf*	91	3	87	8	77	9
Flocoumafen *screen*	85	3	86	8	80	10
Flocoumafen *conf*	86	3	86	11	76	13
Brodifacoum *screen*	83	4	78	12	77	17
Brodifacoum *conf*	83	7	83	12	76	12
Difethialone *screen*	79	3	80	5	82	15
Difethialone *conf*	85	3	84	11	87	6

Residue levels in real sample extracts were interpolated from calibration data generated in the same experimental batch (i.e. standards, matrix/reagent blanks, AQC sample and real samples). Whenever higher level residues were indicated i.e. >>0.1 mg kg^−1^, the higher level standards were ‘retrospectively’ included and a quadratic 8-point calibration curve (0.025–1 mg kg^−1^) was generated and used for quantitation. The utility of this approach was proven by comparison of results obtained following analysis of diluted and original sample extracts that contained high-level residues, using linear and quadratic calibration, respectively, Table 6.

**Table 6:** Comparison of quantitation results of high level bromadiolone residues in diluted and original sample extracts using linear and quadratic calibration, respectively.

Matrix Linear Std. Range (0.0005–0.01 μgml^−1^): **DILUTED**				
AR	Bromadiolone 1		Bromadiolone 2	
MRM	525.2 > 250.10		525.2 > 181.15	
*R^2^*	*0.9999*		*0.9999*	
Sample	μg ml^−1^	mg kg^−1^	μg ml^−1^	mg kg^−1^
Fox/1 100 ml/2 g	0.0069	0.3450	0.0073	0.3650
Fox/2 100 ml/1.35 g	0.0077	0.5704	0.0077	0.5704
Fox/3 200 ml/2 g	0.0080	0.8000	0.0079	0.7900
Dog/1 500 ml/2 g	0.0064	1.6000	0.0062	1.5500

Matrix Quadratic Std. Range (0.0005–0.5 μgml^−1^): **ORIGINAL**				
*R^2^*	*0.9997*		*0.9996*	
Sample	μg ml^−1^	mg kg^−1^	μg ml^−1^	mg kg^−1^
Fox/1 10 ml/2 g	0.0743	0.3715	0.0736	0.3680
Fox/2 5 ml/1.35 g	0.1474	0.5459	0.1452	0.5378
Fox/3 10 ml/2 g	0.1707	0.8535	0.1665	0.8325
Dog/1 10 ml/2 g	0.3744	1.8720	0.3677	1.8385

Measured concentrations in real samples are not corrected for recovery.

Initial gross residue determinations included a top calibration standard of 0.5 μgml^−1^.

The extended calibration range was reduced for routine use to 0.0005–0.2 μg ml^−1^. Exclusion of the 0.5 μg ml^−1^ standard eliminated risk of carry-over and reduced consumption of expensive reference materials without any adverse effect on quantitation of gross residues. Consequently, dilution may still be required but more infrequently.

## Additional information

The environmental impact of legitimate AR use is monitored in many countries as data collected underpins ongoing review, risk-assessment and refinement of the conditions and guidance for use [5]. Monitoring activities can also confirm or refute the mis-use of AR i.e. suspected accidental, negligent or deliberate poisonings of non-target vertebrate wildlife, pets and livestock. In the United Kingdom, surveillance of the impact of rodenticide use is facilitated by the UK’s Wildlife Incident Investigation Scheme (WIIS) which is operated in Scotland by Science and Advice for Scottish Agriculture (SASA) on behalf of the Scottish Government [6]. The active ingredients present in AR products currently approved for use in the UK include; brodifacoum, bromadiolone, coumatetralyl, difenacoum, difethiolone, flocoumafen and warfarin. Chloropacinone and diphacinone are still sought as legacy chemicals and to reveal any illegal use.

**Figure:**
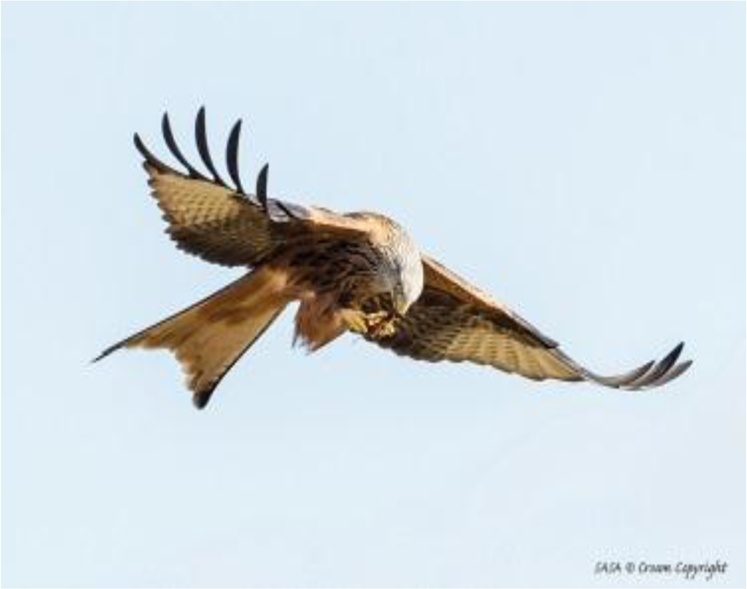

**Fig. 2:**
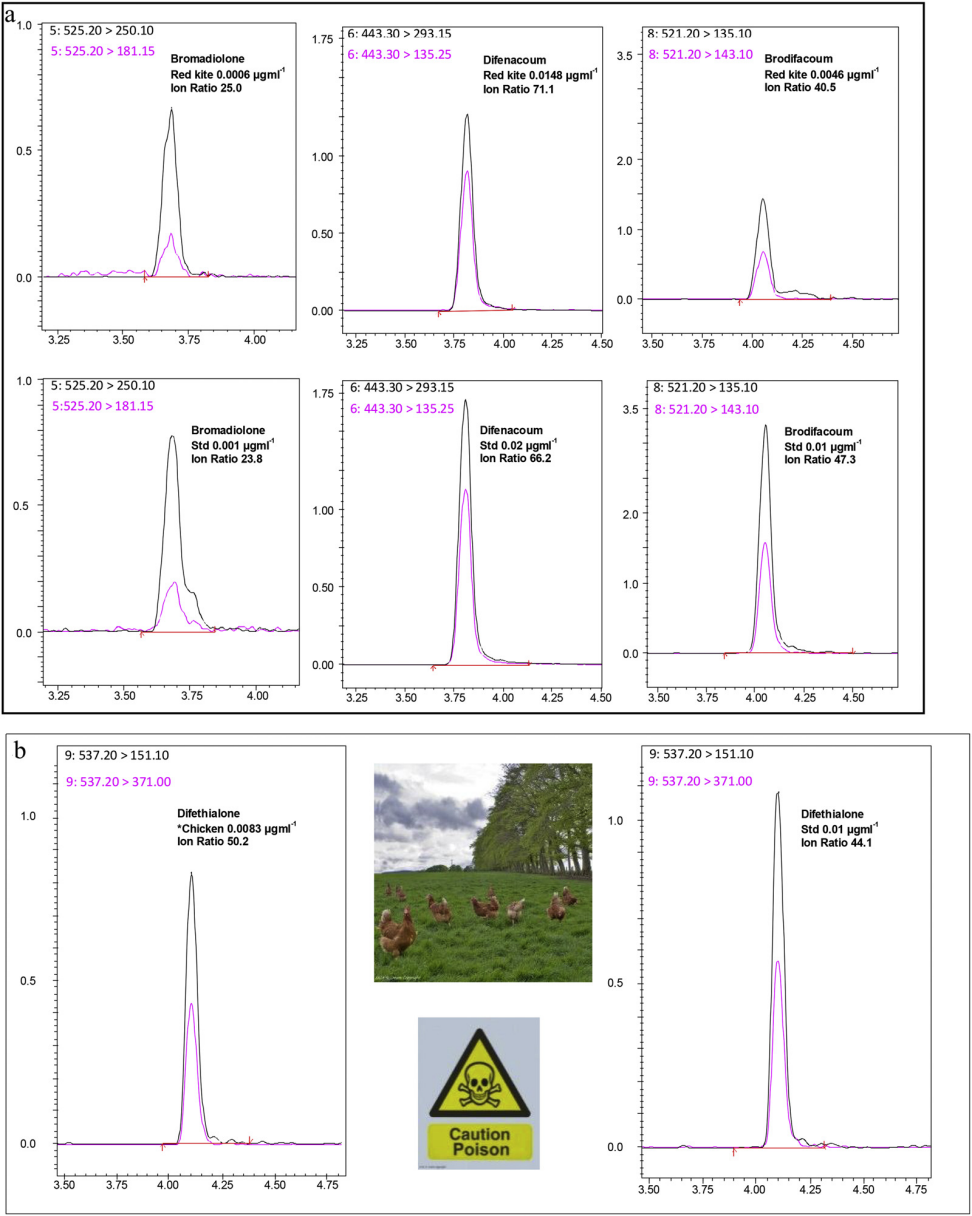
(a) Brodifacoum, bromadiolone and difenacoum residues detected in a red kite (*Milvus milvus*). Screen and confirmation MRMs from liver extract, proximate standards and ion ratio compliance. Fig. 2b, shows data that confirmed (suspected) poisoning of numerous domestic chickens accidentally and fatally exposed to difethialone AR product used at a farm by the owner. (b) Confirmation of accidental difethialone poisoning of domestic chickens (*Gallus gallus domesticus*): Screen, confirmation and proximate standard MRMs *Actual Residue = 0.45 mgkg^−1^ (1:20) dilution required. (For interpretation of the references to colour in this figure legend, the reader is referred to the web version of this article.)

All samples used for validation studies and subsequent routine deployment of the method were submitted as part of WIIS-Scotland surveillance program. The method is also routinely applied, but not (currently) validated, for the determination of multiple-AR residues in a variety of matrices i.e. suspected bait samples, plasma, whole-blood, viscera and unknown substances, contaminated materials/items and formulated products. Fig. 2a and b show typical results obtained following routine application of the method which has significantly improved experimental precision and workflow efficiency. Fig. 2a presents experimental data confirming the presence of bromadiolone, brodifacoum and difenacoum residues in the liver of a red kite, which is a protected species in the UK. Exposure was concluded to be due to consumption of dead or dying rodents.
